# Relationship between the Risk of Gastric Cancer and Adherence to the Mediterranean Diet According to Different Estimators. MCC—Spain Study

**DOI:** 10.3390/cancers13215281

**Published:** 2021-10-21

**Authors:** Laura Álvarez-Álvarez, Facundo Vitelli-Storelli, María Rubín-García, Nuria Aragonés, Eva Ardanaz, Gemma Castaño-Vinyals, Mireia Obón-Santacana, Trinidad Dierssen-Sotos, Dolores Salas-Trejo, Adonina Tardón, José Juan Jiménez Moleón, Juan Alguacil, María Dolores Chirlaque, Beatriz Pérez-Gómez, Marina Pollán, Manolis Kogevinas, Vicente Martín

**Affiliations:** 1 Group of Investigation in Interactions Gene-Environment and Health (GIIGAS), Institute of Biomedicine (IBIOMED), University of León, 24071 León, Spain; lalva@unileon.es (L.Á.-Á.); fvits@unileon.es (F.V.-S.); vicente.martin@unileon.es (V.M.); 2 Consortium for Biomedical Research in Epidemiology & Public Health, CIBERESP, 28029 Madrid, Spain; nuria.aragones@salud.madrid.org (N.A.); me.ardanaz.aicua@cfnavarra.es (E.A.); gemma.castano@isglobal.org (G.C.-V.); dierssent@unican.es (T.D.-S.); salas_dol@gva.es (D.S.-T.); atardon@uniovi.es (A.T.); jjmoleon@ugr.es (J.J.J.M.); alguacil@dbasp.uhu.es (J.A.); mdolores.chirlaque@carm.es (M.D.C.); bperez@iscii.es (B.P.-G.); mpollan@iscii.es (M.P.); manolis.kogevinas@isglobal.org (M.K.); 3 Cancer Epidemiology Section, Public Health Division, Department of Health of Madrid, 28035 Madrid, Spain; 4 Navarra Institute for Health Research (IdiSNA) Navarra Public Health Institute, 31008 Pamplona, Spain; 5 ISGlobal, Centre for Research in Environmental Epidemiology (CREAL), 08036 Barcelona, Spain; 6 Oncology Data Analytics Program (ODAP), Catalan Institute of Oncology (ICO), L’Hospitalet Del Llobregat, 08908 Barcelona, Spain; mobon@idibell.cat; 7 Universidad de Cantabria—IDIVAL, 39005 Santander, Spain; 8 Valencian Breast Cancer Screening Program, General Directorate of Public Health, 46020 València, Spain; 9 Department of Medicine, University of Oviedo, 33003 Oviedo, Spain; 10 Instituto de Investigación Biosanitaria de Granada (ibs.GRANADA), Hospitales Universitarios de Granada, Universidad de Granada, 18012 Granada, Spain; 11 Centro de Investigación en Recursos Naturales, Salud y Medio Ambiente (RENSMA), Universidad de Huelva, Campus Universitario de El Carmen, 21004 Huelva, Spain; 12 Department of Epidemiology, Regional Health Council, IMIB-Arrixaca, Murcia University, 30008 Murcia, Spain; 13 Cancer and Environmental Epidemiology Unit, National Center for Epidemiology, Carlos III Institute of Health, 28029 Madrid, Spain; 14 Cancer and Environmental Epidemiology Unit, Department of Epidemiology and Chronic Diseases, National Center for Epidemiology, Carlos III Institute of Health, 28029 Madrid, Spain; 15 Institute of Global Health (ISGlobal), 08036 Barcelona, Spain

**Keywords:** diet, Mediterranean, feeding behaviour, stomach neoplasms

## Abstract

**Simple Summary:**

Dietary habits are one of the factors that influence the development of gastric cancer and, although it has been seen that the Mediterranean diet has a protective effect on this type of cancer, there are different indexes to assess the degree of adherence to this dietary pattern; this implies differences in the results obtained in the reduction of risk. The aim of this work was to assess the effect of adherence to the Mediterranean diet, measured with five different indexes, on the risk of gastric cancer.

**Abstract:**

The aim was to assess the effect of adherence to the Mediterranean Diet, measured with five different indexes, on the risk of gastric cancer. Data come from the multicase-control study MCC—Spain, which included 354 gastric cancer cases and 3040 controls with data on diet. We used five indexes to evaluate adherence to the Mediterranean diet and assess the association between each pattern with the risk of gastric cancer, using multivariate logistic regression. The analyses were performed for the whole set of gastric cancer cases, by anatomical location (cardia and non-cardia) and by histological type (intestinal and diffuse). According to the used index, a high adherence protects one from gastric cancer (between 48% (aOR = 0.52; CI 95% = 0.28–0.94) and 75% (aOR = 0.25; CI 95% = 0.12–0.52)), from non-cardia (between 48% (aOR = 0.52; CI 95% = 0.36–0.75) and 65% (aOR = 0.35; CI 95% = 0.23–0.52)), and from the intestinal type (between 41% (aOR = 0.59; CI 95% = 0.36–0.95) and 72% (aOR = 0.28; CI 95% = 0.16–0.50)), but not from the diffuse type. In conclusion, high adherence to a Mediterranean diet pattern is a protective factor for the risk of gastric cancer, with greater adherence leading to greater protection.

## 1. Introduction

Gastric cancer is a global public health problem. According to the GLOBOCAN report, in 2018, there were 1,033,700 new cases and 782,700 deaths from this cause worldwide. It ranks first in malignant tumors of the gastrointestinal tract, sixth in frequency, and second in cancer deaths [1].

Several authors suggest that dietary habits may be of great importance in this type of cancer [2,3,4], and the World Cancer Research Fund (WCRF) report states that high-salt diets, smoking, nitrates and red and processed meats are associated with an increased risk of gastric cancer. In addition to all of this, it is specified that a consumption of three or more alcoholic beverages daily is associated with an increased risk [5]. Other studies have observed that dietary habits such as high fat intake, fried foods and aflatoxins, and low fruit and vegetable intake are also associated with an increased risk [6].

However, the behavior of cancer on a diet basis cannot be explained only by isolated components because people do not eat food independently, but as part of a specific food pattern that, in addition to food, it establishes the proportions and quantities in which these are ingested [7].

In recent years, the study of these patterns has been increasing and the results, with regard to cancer, point to a detrimental effect of the Western diet (high content of red and processed meats, soft drinks, precooked meals, and fatty dairy) and to a protective effect of the Mediterranean diet (high consumption of vegetable products, legumes, fish, whole grains, and olive oil) [8,9].

The latter is one of the dietary patterns with more scientific evidence published regarding its beneficial health effects. A high adherence to the Mediterranean diet has being associated with the prevention of certain diseases such as diabetes, cardiovascular disease, certain types of cancer, metabolic syndrome, or neurodegenerative diseases, among others [10].

There are different studies and reviews that treat the association between gastric cancer and adherence to the Mediterranean diet and, although greater adherence has been associated with a lower risk of this type of cancer, there is no homogeneity among the used indexes [11,12,13,14,15,16]. Thus, the results in terms of risk reduction also vary.

Although most adherence rates to the Mediterranean diet are based on those created by Trichopoulou et al. [17], there are large differences between them. Score scales are not always the same and statistical parameters also vary (mean, medium, or tertiles); there are also differences among the food or food groups included in each index and in the value of each of the components (positive or negative) [18]. Furthermore, it should be noted that there are indexes where the score is based on the distribution observed in the sample and others based on previously established independent criteria [19].

For this reason, different studies and reviews question the correlation between the different adherence rates to the Mediterranean diet and address the need to reach an agreement and establish a single index [18,19,20].

Therefore, in this work, we focus on five different indexes or methodologies (two of them based on previously defined criteria and three based on sample distribution) to be able to assess the association between gastric cancer and adherence to the Mediterranean diet.

## 2. Materials and Methods

The MCC—Spain project database (www.mccspain.org, accessed on 6 April 2021) was used to carry out this work. MCC—Spain is a population-based, multi-centric, case-control study aimed at evaluating the influence of environmental exposures and genetic factors in five of the most important tumors in the Spanish population: breast, prostate, colorectal, gastric, and chronic lymphocytic leukemia. The project was carried out between September 2008 and December 2013 in 12 Spanish provinces: Asturias, Barcelona, Cantabria, Girona, Granada, Guipúzcoa, Huelva, León, Madrid, Murcia, Navarra, and Valencia [21].

MCC—Spain inclusion criteria indicated that participants had to be between 20 and 85 years old, had been living in the study area for at least 6 months before diagnosis, and were able to answer the questionnaire. Cases of GC were incidents, with histologically confirmed diagnosis (C16, D00.2), with no history of the disease, and that had been diagnosed during treatment. Controls were randomly selected from the lists of general doctors in the area of the hospitals where the cases were recruited, and were often matched by age, sex, and region [21]. Specifically, for gastric cancer, 459 histologically confirmed cases and 3440 controls were recruited, with a response rate of 57% and 53%, respectively [12].

Table 1 shows the characteristics of the selected sample.

The MCC—Spain protocol was approved by each of the Ethics Committees of the participating institutions. All participants were informed of the objectives of the study and signed an informed consent.

In order to assess the participants’ usual dietary intake, a semi-quantitative food frequency questionnaire (FFQ) of 140 items was provided to each [22]. This was a modified version of a questionnaire validated previously [23] to include typical foods from each region. In addition, the consumed portions of each food and the type of cooking were specified. The FFQ was self-administered and mailed back or completed during an interview with a 88% overall response rate [21].

In our work, we used five indices to assess adherence to the Mediterranean diet; that is, Mediterranean Diet Score (MDS) [17], relative Mediterranean Diet (rMED) [24], alternative Mediterranean Diet (aMED) [25], Dietary Score (DS) [26], and literature-based adherence score to Mediterranean diet (MEDI-LITE) [27]. In all of them, a higher score indicates greater adherence to the Mediterranean diet:

**MDS**: With a rank range from 0 to 9. It includes nine food and nutrient groups and uses the median to assign scores.

**rMED**: It also considers nine food groups. The score ranges from 0 to 18 and uses tertiles to assign the scores.

**aMED**: Like MDS, the score of this index ranges from 0 to 9, uses the median to assign scores, and includes nine food and nutrient groups (although with differences from the first).

**DS**: Includes 11 food groups, its classification ranges from 0 to 55, and it uses previously set criteria to assign the scores.

**MEDI-LITE**: Includes nine food groups, its classification ranges from 0 to 18, and it also assigns scores according to a previously defined scale.

In Table 2 and Table 3, adapted from the work of Olmedo-Requena et al. [19], the summary information for the above five indexes is displayed. In the first three indexes, scores are established based on the sample distribution, and in the last two according to fixed criteria defined previously.

To describe the characteristics of the participants, mean and standard deviations (SD) were calculated for quantitative variables and relative frequency distributions for qualitative variables.

Participants were classified as low, medium, and high adherence to the Mediterranean diet according to the cut-off points established in the selected indices: 0–3, 4–6, and 7–9 for MDS and aMED, respectively; 0–7, 8–13, and 14–18 for rMED; tertile 1, 2, or 3 for DS; and <9, 9–11, and >11 for MEDI-LITE.

The association between each dietary pattern and gastric cancer risk was evaluated using logistic regression. Odds ratios were calculated with their 95% confidence interval for all gastric cancer cases, for each location (cardia and non-cardia), and for each histological subtype (intestinal and diffuse). All models were adjusted for sex, age, and education as fixed effects and area of residence as a random effect term.

The calculation of further adjusted odds ratios took into account the variables sex, age, education, family history of gastric cancer (first degree), tobacco status, total energy consumed, BMI (the year before diagnosis), consumption of NSAIDs, and total time of physical activity as fixed terms and area of residence as a random effects variable.

## 3. Results

A total of 459 cases and 3440 controls were recruited. Of the total number of participants selected, information on food consumption was obtained only in 354 cases (most of them adenocarcinomas) and 3040 controls. In Table 4, we can see how, in general, the scores obtained are higher in controls than in cases, indicating greater adherence to the Mediterranean diet in the first group, obtaining statistically significant differences in four of the five indexes used.

**Figure 1:**
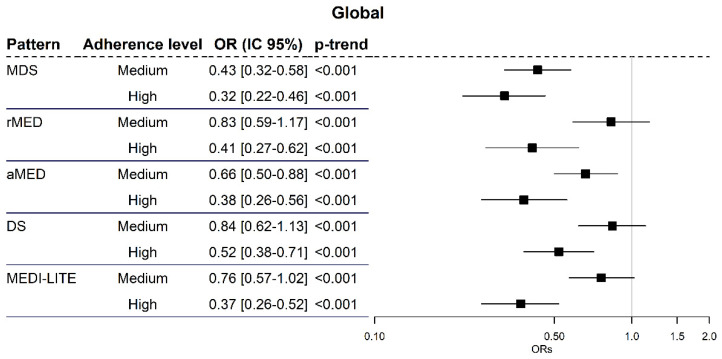
Odds ratios of risk of gastric cancer according to adherence level to various rates compared with low adhesion.

A statistically significantly decreased gastric cancer risk was observed for those participants with high adherence compared with those with low adherence, regardless of the studied score: between 48% for DS (aOR = 0.52; 95% CI = 0.38–0.71) and 68% for MDS (aOR = 0.32; 95% CI = 0.22–0.46). In addition, medium adherence to the Mediterranean diet protects from gastric cancer from low adherence between 16% for DS (aOR = 0.84; 95% CI = 0.62–1.13) and 57% for MDS (aOR = 0.43; CI 95% = 0.32–0.58), and although, in this case, the differences are not always statistically significant, a clear trend is observed (Figure 1).

**Figure 2:**
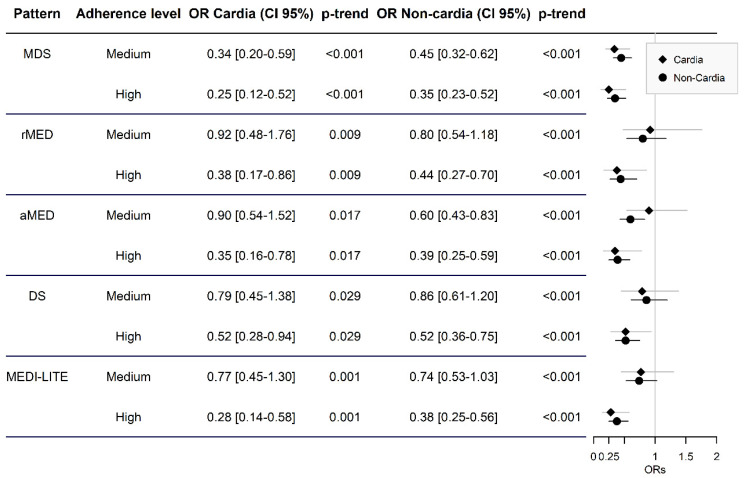
Odds ratios of risk of gastric cardia and non-cardia cancer according to adherence level to various indexes compared with low adhesion.

As for locations, in Figure 2, we can observe how, for cardia cancer, with all the indexes used, a high adhesion limits the risk of gastric cancer to the Mediterranean diet between 48% for DS (aOR = 0.52; CI 95% = 0.28–0.94) and 75% for MDS (aOR = 0.25; CI 95% = 0.12–0.52), opposite to the low adhesion. In the case of medium adhesion, although a risk reduction is also observed by 8% for rMED (aOR-0.92; 95% CI 0.48–1.76) and 66% for MDS (aOR-0.34; 95% CI 0.20–0.59), only statistically significant associations were obtained in one of the indexes (MDS).

As for gastric non-cardia cancer, in Figure 2, we observed how, as in the previous case, a high level of adherence to the Mediterranean diet reduces the risk of gastric cancer compared with a low level by 48% for DS (aOR = 0.52; 95% CI 0.36–0.75) and 65% for MDS (aOR = 0.35; 95% CI 0.23–0.52). For medium adherence, risk reduction varies between 14% for DS (aOR = 0.86; 95% CI = 0.61–1.20) and 55% for MDS (aOR = 0.45; 95% CI = 0.32–0.62), but only statistically significant associations were found in two of the indexes (MDS and aMED).

**Figure 3:**
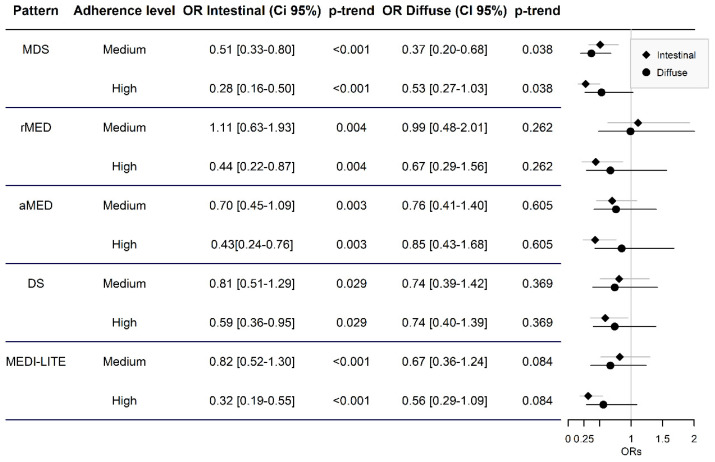
Odds ratios of risk of intestinal and diffuse adenocarcinoma according to adherence level to various indexes compared with low adhesion.

As for the different histological subtypes of adenocarcinoma, in Figure 3, we see how, in the intestinal type, high adherence reduces the risk of gastric cancer by 41% for DS (aOR = 0.59; 95% CI = 0.36–0.95) and 72% for MDS (aOR = 0.28; CI 95% = 0.16–0.50) versus low adherence, with statistically significant results in all the indexes analyzed. For medium adherence, it is only statistically significantly reduced for MDS. Finally, no statistically significant associations were observed for high adherence diffuse gastric cancer risk.

## 4. Discussion

The results obtained in our work show that adherence to a Mediterranean diet pattern, assessed through five different indexes, is a protective factor for the risk of gastric cancer, obtaining greater protection with higher adherence to this dietary pattern.

According to the index used, high adherence protects from gastric cancer between 48% (DS) and 68% (MDS) against low adherence, showing statistically significant differences in the five indexes analyzed. In the case of medium versus low adherence, despite observing decreasing risk in all indexes (between 16% and 57%), the results were not always statistically significant.

Our results are in line with those presented by Stojanovic et al. [28] and Buckland et al. [16], among others. In the first case, high adherence to the Mediterranean pattern, measured through the MEDI-LITE score, was associated with a 30% reduction in gastric cancer risk (OR: 0.70; 95% CI: 0.61–0.81). In Buckland et al., using the rMED index, the reduction was also significant (HR 0.67; 95% CI 0.47–0.94).

This also coincides with the results of the meta-analysis by Du et al. [29], in which, for case-control studies, high adherence scores were associated with an average risk reduction of 58% (OR 0.42; 95% CI 0.20–0.86).

As for the different locations of gastric cancer, in our work, we found a statistically significantly reduced relative risk for high adherence versus low adherence in both cardia (between 48% and 75%) and non-cardia cancer (between 48% and 65%). These results are partly in line with those obtained by Schulpen et al. [11], because a high adherence to aMED (alcohol-free) was associated with a reduced risk in both locations, although it was only significant in men.

On the other hand, it contrasts with the results obtained by Buckland et al. [16], in which high adherence was associated with a significant reduction in the risk of gastric cardia cancer (OR 0.45; 95% CI 0.21–0.91), but not with non-cardia cancer. It also does not coincide with Li et al. [30], in which no protective effect was found, neither for cardia nor non-cardia gastric cancer.

Finally, in relation to the different histological types, we found a significant inverse association for high adhesion in the intestinal type (between 41% and 72%), but not in the diffuse type. To the best of our knowledge, the association between the adherence to the Mediterranean diet and histological types has only been evaluated by Buckland et al. [16] and, in this case, although risk reduction was observed in both types, the results were not significant.

Numerous health benefits have been derived from following a Mediterranean diet: cardiovascular, higher life expectancy, or lower risk of certain types of cancer, among others [10,31,32], and this has resulted in a wide variety of indexes to assess adherence to this dietary pattern [20].

These indexes should be comparable as the final objective is the same; however, as different publications show, the correlations between them are not always as high as one would expect [18,19]. This can be explained by the lack of common criteria when designing indexes: type of food included, range of scores, or criteria for designing the index.

Among the indexes selected in this paper, we see the following differences. First of all, they do not take into account the same foods or nutrients: there are indexes that include all types of meat (MDS and rMED) and others that only take into account red meat (aMED); all indexes include dairy, except aMED; and there is also a significant difference in assessing fat intake (MDS and aMED value the ratio between monounsaturated/saturated fats, while rMED, DS, and MEDI-LITE value the amount of olive oil consumed). Secondly, the score range assigned to each index is different. While MDS and aMED use a scale ranging from 0 to 9, the DS index is from 0 to 55. Moreover, the criteria chosen to assign the scores do not match. rMED ranks by tertiles; MDS and aMED use the median; and DS and MEDI-LITE use a fixed previously established score. Finally, there are indexes that are dependent on the sample studied and others that are not. While DS and MEDI-LITE set sample-independent fixed criteria, MDS, aMED, and rMED are calculated based on sample distribution.

For this reason, although all indices measure adherence to the Mediterranean diet pattern, we note that there are important differences between them that can compromise reliability. This is why different authors see the need to reach an agreement to use a common or homogeneous index that guarantees greater accuracy in assessing adherence to this dietary pattern [18,21,33].

### Strengths and Limitations

Although there are numerous publications on the relationship of the Mediterranean diet and gastric cancer, to date, no work has been published to evaluate the effect of adherence to this dietary pattern in the Spanish population, using five different indexes and taking into account the location and histological type of cancer. The results were consistent between gastric cancer subtypes and most of them remained statistically significant in stratified analyses, suggesting that our findings were unlikely entirely due to chance.

The reason for analyzing this association with different indexes was because of the concordance problems existing between the different ways of assessing adherence to the Mediterranean diet, which several authors have already stated [18,19].

Another strength of this study is the inclusion of participants from 12 different provinces, which guarantees a representative sample and gives it greater external validity. Our study has several limitations. Firstly, the main limitation of the work is the possibility of recall bias, as the information collected is reported by the participant himself. In addition, the dietary information analyzed is that relating to consumption only from the previous year and is based on an estimate of consumption, which may have an impact on the calculation of adherence.

Secondly, there may also be some confounding factor that has not been taken into account. Differences in the distribution of some characteristics of the cases and controls included in our study do exist, and participants with a higher socioeconomic or educational status were more likely to participate in the study, especially among controls. In this sense, although we controlled for potential confounders, residual confounding cannot be entirely ruled out.

Finally, the size of our sample has not allowed us to analyze the possible interaction between adherence to the Mediterranean diet and sex according to different locations or histological types of the tumor.

## 5. Conclusions

Our results support the protective role of adherence to a Mediterranean diet pattern for gastric cancer risk, and this protection seems to increase with greater adherence. Although there is variation in the results obtained with the different indexes, in the five cases, statistically significant trends in risk reduction ranged from 48% to 68% for high adherence and from 16% to 57% for medium adherence.

This statistically significant protective association was also observed using the five indexes for both gastric cardia and non-cardia gastric cancer. The protection of high versus low adherence oscillated between 48% and 75% in the first case and between 48% and 65% in the second case depending on the index used.

With regard to the different histological types, with the five indexes, we found a significant protective effect between 41% and 72% for high adherence versus low adherence in the intestinal type. However, in the diffuse type, we only found a significant protective effect with one of the indexes analyzed.

As mentioned above, it would be interesting, for future research, to standardize the criteria of the indexes used in order to strengthen the evidence of the relationship between the Mediterranean diet and the improvement in health.

## Figures and Tables

**Table 1:** General characteristics of cases and controls.

Variables	Cases		Controls		*p*-Value
	*n*	%	*n*	%	
Sex					<0.001
Men	308	67.1	1892	55.0	
Women	151	32.9	1548	45.0	
Age					<0.001
<59	140	30.5	1068	31.1	
60–74	184	40.1	1784	51.9	
>74	135	29.4	588	17.1	
Educational level					<0.001
Less than primary education	138	30.1	651	18.9	
Primary education	177	38.6	1153	33.5	
Secundary education	96	20.9	951	27.7	
University	48	10.5	685	19.9	
Area of residence					<0.001
Asturias	14	3.1	194	5.6	
Barcelona	100	21.8	1033	30.0	
Cantabria	24	5.2	353	10.3	
Granada	5	1.1	120	3.5	
Huelva	15	3.3	155	4.5	
León	123	26.8	440	12.8	
Madrid	110	24.0	731	21.3	
Murcia	1	0.2	7	0.2	
Navarra	53	11.6	271	7.9	
Valencia	14	3.1	136	4.0	
First-degree family history					<0.001
Yes	71	15.5	208	6.1	
No	363	79.1	3020	87.8	
Unknown	25	5.5	212	6.2	
Smoking habit					0.184
Never	186	40.5	1507	43.8	
Former	161	35.1	1204	35.0	
Current	111	24.2	711	20.7	
Unknown	1	0.2	18	0.5	
BMI					0.012
<25 kg/m^2^	112	24.4	1037	30.2	
25–29 kg/m^2^	169	36.8	1155	33.6	
≥30 kg/m^2^	99	21.6	586	17.0	
Unknown	79	17.2	662	19.2	

**Table 2:** Characteristics of the different adherence indexes to the Mediterranean diet.

Food Groups	MDS (0–9 Points)			rMED (0–18 Points)			aMED (0–9 Points)		
		Scoring Criteria			Scoring Criteria			Scoring Criteria	
Vegetables	All	≥median	g/day	All (except potatoes)	Tertile	g*1000 kcal/day	All (except potatoes)	>median	rations/day
Legumes	All	≥median	g/day	All	Tertile	g*1000 kcal/day	All	>median	rations/day
Fruit	Included nuts	≥median	g/day	Included nuts	Tertile	g*1000 kcal/day	Included juices	>median	rations/day
Nuts							All	>median	rations/day
Cereals	All	≥median	g/day	All	Tertile	g*1000 kcal/day	Only whole grains	>median	rations/day
Fish	Included seefod	≥median	g/day	Included seafood and frozen fish	Tertile	g*1000 kcal/day	Included seefood	>median	rations/day
Meat	All	≤median	g/day	All	Tertile (reverse)	g*1000 kcal/day	Only red and processed meats	<median	rations/day
Dairy products	Milk, cheese and yogurt	≤median	g/day	Included cream and milk fat	Tertile (reverse)	g*1000 kcal/day			
Fats	Ratio MF/SF	≥median	g/day	Olive oil	Tertile	g*1000 kcal/day	Ratio FM/FS	>median	
Alcohol	All types	M: 10–50; W: 5–25	g/day	All types	M: 10–50; W: 5–25	g/day	All types	M: 10–25; W: 5–15	g/day

**Table 3:** Characteristics of the different adherence indexes to the Mediterranean diet.

Food Groups	DS (0–55 Points)			MEDI-LITE (0–18 Points)		
		Scoring Criteria			Scoring Criteria	
Vegetables	All		rations/month	Not especified	<1p (0); 1–2.5p (1); >2.5p (2)	portion/day (p = 100 g)
Potatoes	All	0 -> 0 points	rations/month			
Legumes	All	1–4 -> 1 point	rations/month	Not especified	<1p (0); 1–2p (1); >2p (2)	portion/week (p = 70 g)
Fruit	All	5–8 -> 2 points	rations/month	Not especified	<1p (0); 1–2p (1); >2p (2)	portion/day (p = 150 g)
Nuts		9–12 -> 3 points				
Cereals	Unrefined	13–18 -> 4 points	rations/month	Not especified	<1p (0); 1–1.5p (1); >1.5p (2)	portion/day (p = 130 g)
Fish	All	>18 -> 5 points	rations/month	Not especified	<1p (0); 1–2.5p (1); >2.5p (2)	portion/week (p = 100 g)
Meat	Red	Reverse	rations/month	Not especified	<1p (2); 1–1.5p (1); >1.5p (0)	portion/day (p = 80 g)
Poultry	All	Reverse	rations/month			
Dairy products	Milk, cheese and yogurt (whole)	Reverse	rations/month	Not especified	<1p (2); 1–1.5p (1); >1.5p (0)	portion/day (p = 180 g)
Fats	Olive oil	never:0p, hardly ever:1p, ≤1:2p, 1–3: 3p, 3–5: 4p, daily: 5	use	Olive oil	Occasional (0) Frequent (1) Usual (2)	Frequency of use
Alcohol	All types	<300: 5, 300–399: 4, 400–499: 3, 500–599: 2, 600–699: 1, >700: 0	ml/day	All types	<1AU (1); 1–2 AU (2); >2 AU (0)	Alcohol unit/day (1AU = 12 g)

**Table 4:** Average MD adherence score by group for each index used.

Index	Cases		Controls		*p*
	Mean	SD	Mean	SD	
MDS (Trichopoulou et al.) [17]	3.86	1.51	4.27	1.67	<0.001
rMED (Buckland et al.) [24]	8.99	3.11	8.79	3.26	0.28
aMED (Fung et al.) [25]	3.63	1.69	4.04	1.77	<0.001
DS (Panagiotakos et al.) [26]	33.67	4.24	34.55	4.29	0.0003
MEDI-LITE (Sofi et al.) [27]	8.83	2.20	9.51	2.18	<0.001

MDS = Mediterranean Diet Score; rMED = Relative Mediterranean Diet; aMED = Alternative Mediterranean Diet; DS = Dietary Score; MEDI-LITE = literature-based adherence score to Mediterranean diet.

## Data Availability

Protocol to the MCC—Spain study https://www.mccspain.org/; protection of data, enlisted in the Data Protection Agency (Number 2102672171).
